# Self-rated quality of life and school performance in relation to helminth infections: case study from Yunnan, People's Republic of China

**DOI:** 10.1186/1756-3305-3-61

**Published:** 2010-07-23

**Authors:** Kathrin Ziegelbauer, Peter Steinmann, Hui Zhou, Zun-Wei Du, Jin-Yong Jiang, Thomas Fürst, Tie-Wu Jia, Xiao-Nong Zhou, Jürg Utzinger

**Affiliations:** 1 Department of Epidemiology and Public Health, Swiss Tropical and Public Health Institute, P.O. Box, CH-4002 Basel, Switzerland; 2 University of Basel, P.O. Box, CH-4003 Basel, Switzerland; 3 National Institute of Parasitic Diseases, Chinese Center for Disease Control and Prevention, Shanghai 200025, People's Republic of China; 4 Helminthiasis Division, Yunnan Institute of Parasitic Diseases, Pu'er 665000, People's Republic of China

## Abstract

**Background:**

Expert opinion-derived disability weights are widely employed for estimating the global burden of diseases and injuries. For chronic diseases such as soil-transmitted helminthiasis and schistosomiasis, it has been suggested that a patient-based quality of life (QoL) approach should be considered for a more accurate appraisal of disability weights.

**Methods and Results:**

We carried out a cross-sectional survey and assessed the prevalence and intensity of soil-transmitted helminth infections as well as self-rated QoL indicators among 252 students attending grades 5-8 in two schools (Bulangshan and Pu'er) in Yunnan province, People's Republic of China. Each student provided a single stool sample, which was subjected to duplicate Kato-Katz thick smear readings and a single FLOTAC examination for parasitological diagnosis. Prevalence rates for hookworm, *Trichuris trichiura *and *Ascaris lumbricoides *were high in Bulangshan (75.9%, 70.0% and 68.2%), while the respective prevalence rates in Pu'er were 66.9%, 56.5% and 9.2%. Students were interviewed with two standardised questionnaires, the EuroQoL-5 Dimensions (EQ-5D) and ShortForm-12 (SF-12) Health Survey. Impairment in any of the five dimensions of the EQ-5D was reported by 87% of the students. However, no clear differences could be observed between individuals with and those without helminth infections, and there were discrepancies between the two schools. A multivariate logistic regression model revealed no differences between students with varying infection status in the domains of the SF-12 (odds ratio close to 1.0). Somewhat more pronounced, yet not statistically significant differences were observed when end-of-school-term marks were compared with students' helminth infection status: infected individuals had lower marks in Chinese, English and mathematics, but not in sports, compared to their helminth-free counterparts.

**Conclusions:**

Our results point to unresolved issues and challenges regarding the cultural appropriateness of the widely used standard QoL questionnaires. Hence, new research is needed to further develop these instruments and to validate them in connection with chronic parasitic diseases.

## Background

According to the global burden of disease (GBD) study published in 1996, soil-transmitted helminthiasis (infections with the nematodes *Ascaris lumbricoides*, *Trichuris trichiura *and the two hookworm species *Ancylostoma duodenale *and *Necator americanus*) causes the loss of 5 million disability-adjusted life years (DALYs) every year [1]. However, other sources put forth an 8-fold higher estimate, i.e. 39 million DALYs [2,3]. The DALY estimates utilised in the initial GBD study were established by expert committees based on disease scenarios that arguably failed to reflect the full spectrum of morbidities, notably chronic conditions [4,5]. While an infection with one or several soil-transmitted helminth species seldom causes death, chronic infections with moderate or heavy worm burdens result in considerable morbidity, including stunting, wasting, anaemia and impaired physical and mental development in children [6,7]. Because of the mainly subtle morbidity, the long-term chronicity and the high prevalence of multiple species infections, it is difficult to estimate the specific burden due to the different soil-transmitted helminth species [8].

Another approach for assessing the burden related to a particular condition is based on patient-based quality of life (QoL) interviews. Two types of instruments are available: (i) generic instruments, which include general health profiles; and (ii) specific instruments, which focus on problems associated with individual diseases, patient groups or areas of function. Both types are multifactorial constructs and assess the individual's perception of physical, mental and social functioning [9,10]. Two of the most widely used generic QoL questionnaires are the EuroQoL-5 Dimension (EQ-5D) and the ShortForm-12 (SF-12) questionnaire from QualiMetrics [11,12]. Both tools were developed based on 'western' concepts, but they have been validated and used in other societies, including the People's Republic of China (P.R. China) [13,14]. Recently, these tools were utilised for estimating age-specific disability weights for chronic schistosomiasis japonica [15] and echinococcosis [16] in P.R. China. The EQ-5D assesses the patients' QoL in five dimensions (i.e. mobility, self-care, usual activities, pain/discomfort and anxiety/depression), each at three levels of increasing impairment (i.e. no problem, moderate problems and extreme problems). The questionnaire also includes a visual analogue scale (EQ-VAS) where the respondent is asked to rate his or her general health status from best imaginable (100) to worst imaginable (0) [11]. The SF-12 tool consists of 12 questions and the results are combined into eight domains: physical health (PH), role-physical (RP), bodily pain (BP), general health (GH), vitality (V), social functioning (SF), role-emotional (RE) and mental health (MH). In addition, two component scores, the physical component summary (PCS) and the mental component summary (MCS), are evaluated [12].

The purpose of the present study was to assess the self-rated QoL among students in two schools in an area of Yunnan province, P.R. China where soil-transmitted helminths are highly endemic, and to compare questionnaire results with parasitological findings, placing emphasis on helminth infection status, infection intensity, and single versus multiple species helminth infections. In addition, students' end-of-school-term marks in Chinese, English, mathematics and sports were analysed and related to the students' helminth infection status.

## Methods

### Study area and population

We carried out a cross-sectional survey in two schools in southern Yunnan province, P.R. China, from May to June 2009. One school is located in Bulangshan village (geographical coordinates: 21°37'00 N latitude and 100°24'00 E longitude; altitude: 1,490 m above sea level) in Menghai county, Xishuangbanna prefecture. Bulangshan is a village situated in a hilly area and mainly inhabited by the Bulang ethnic minority. Studies conducted in this area showed high prevalence rates of soil-transmitted helminth infections [17,18]. The other school is based in the outskirts of Pu'er (formerly: Simao) city in Pu'er prefecture at 22°46'00 N latitude and 101°04'60 E longitude at an altitude of 1,550 m above sea level. In Pu'er, for several years now, there has been annual school-based deworming, usually administered in January. To our knowledge, no regular deworming has been implemented in Bulangshan.

### Questionnaires and parasitological procedures

The headmasters of both schools were informed about the objectives and procedures of the study. Subsequently, the headmasters introduced the study team to the teachers and students. Each student attending nine randomly selected classes of grades 5 to 8 was eligible to participate in the study and was assigned a unique identification (ID) number. Plastic containers were labelled with the name and ID number, and the students were invited to submit one stool sample in the following week.

Between 08:00 and 14:00 h of the following days, stool samples were collected and transferred to the laboratory of the local hospital or parasitological institute. Duplicate Kato-Katz thick smears were prepared from each sample, using 41.7 mg templates [19]. After a clearing time of 30-45 min, the slides were examined by experienced laboratory technicians. For quality control, the independently obtained readings were compared, and slides were re-read if there were inconsistencies. In addition, a 1 g sub-sample of each stool specimen was weighted and stored in a Falcon tube containing 9 ml 5% formalin solution for subsequent FLOTAC examination. The preserved samples were transferred to the Center for Disease Control and Prevention (CDC) in Wuhu, Anhui province, and processed according to a standard protocol developed by Cringoli et al. [20].

All students who had submitted at least one stool sample were invited to answer a questionnaire. First, one fieldworker explained the purpose of the interview. After all upcoming questions had been answered, questionnaires were handed out and the staff read each question aloud and provided further explanations if needed. The questionnaire consisted of two parts. The first section contained general questions (demography, socio-economic variables), while the second section was designed to assess the self-rated QoL. It included questions pertaining to: (i) health-related infrastructure and behaviour; (ii) self-reported signs and symptoms; and (iii) the two standard questionnaires for assessing health-related QoL, EQ-5D (EuroQoL) and an adapted version of the SF-12. The EQ-5D was already available in Chinese; the rest of the questionnaire was translated into Mandarin Chinese. The questionnaire was pre-tested among 30 children visiting grade 4 in a school near Pu'er city. For quality control, all questionnaires were checked for completeness upon return, and missing or implausible answers were reviewed together with the student.

Students' end-of-school-term marks in Chinese, English, mathematics and sports were obtained from the responsible teachers.

### Data management and statistical analysis

The target sample size was 210 students, based on the following assumptions: prevalence of soil-transmitted helminth infection (65%), a standard deviation of 25%, a difference of the means in the dimensions of the questionnaires between infected and non-infected students of 10, a confidence level of 95% and a power of 80%. A 10% safety margin and an assumed 20% drop out rate translated into a target enrolment number of 276 individuals.

Data were double-entered and cross-checked using EpiData version 3.1 (EpiData Association; Odense, Denmark). All statistical analyses were performed in STATA version 9.1 (StataCorp LP; Texas, USA). Helminth infection status and multiparasitism were determined based on the pooled results of duplicate Kato-Katz thick smears and the FLOTAC test. The arithmetic mean number of eggs counted on the two Kato-Katz slides was multiplied by a factor of 24 to obtain eggs per gram of stool (EPG). Infection intensities for *A. lumbricoides*, hookworm and *T. trichiura *were stratified according to cut-offs put forward by the World Health Organization (WHO) [21]. The EQ-5D and SF-12 were analysed as recommended by EuroQoL [11] and QualiMetrics [22].

Pearson's χ^2^-test and Fisher's exact test, as appropriate, were used to assess the associations between various infection categories, namely infection status (presence or absence of a specific helminth infection), infection intensity (no, light, moderate/heavy), single and multiple helminth species infection, and demographic variables as well as the five dimensions of the EQ-5D. The arithmetic mean of the EQ-VAS, the domains of SF-12 and the end-of-school-term marks in Chinese, English, mathematics and sports were calculated for the different helminth infection categories and their relationship assessed using the Kruskal-Wallis test. Questionnaires with missing data were excluded from the respective analyses. Logistic regression was used to control for confounding (sex and age). Since there was a strong interaction between the two schools, all results were calculated for each school separately.

### Ethical considerations and treatment

The study was integrated into ongoing epidemiological investigations and control activities against soil-transmitted helminthiasis. Ethical clearance had been granted by the academic board of the National Institute of Parasitic Diseases, China CDC. Written informed consent was obtained from the headmasters of both schools prior to data collection. An information sheet describing the study was available for all participants.

At the end of the study, all students were treated free-of-charge with a single oral dose of albendazole (400 mg).

## Results

### Compliance and study cohort

**Figure 1:**
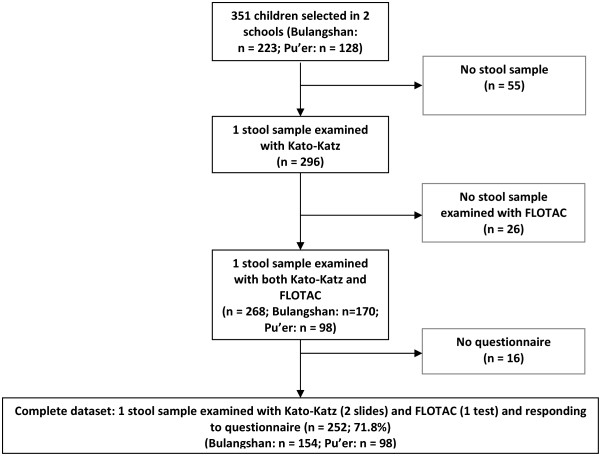
**Compliance and study cohort for the assessment of helminth infection and self-rated QoL in two schools in Yunnan province, P.R. China**. All children with one stool sample examined with the Kato-Katz (2 slides) and FLOTAC technique (1 test) and complete questionnaire results were included in the final analysis.

A total of 351 individuals were invited to participate in the study; 223 in Bulangshan and 128 in Pu'er. Figure 1 shows that 55 students did not provide any stool sample, 26 stool samples were of insufficient quantity to perform duplicate Kato-Katz thick smears plus a FLOTAC examination, and 16 students were absent during the questionnaire survey. Hence, complete datasets, i.e. at least one stool sample subjected to duplicate Kato-Katz thick smear readings and a single FLOTAC and complete questionnaire results, were available from 252 students (154 in Bulangshan and 98 in Pu'er). The overall compliance was 71.8%. The age of the participants ranged from 12 to 27 years, which is typical for the current setting, where many male students first attend temple schools before enrolling for regular schooling. The median age was 14 years and 80% of the participants were between 12 and 14 years old. Farming was the main source of family income, as reported by 89.0% of the students from Bulangshan and 55.1% of the students from Pu'er. Other sources of income were teacher or civil servant (5.1% and 8.2%, respectively), having their own business (3.9% and 12.2%, respectively) or local or migrant worker (2.0% and 24.5%, respectively).

### Soil-transmitted helminth infections

The pooled results from the duplicate Kato-Katz thick smears and a single FLOTAC revealed high prevalence rates of hookworm (75.9%), *T. trichiura *(70.0%) and *A. lumbricoides *infections (68.2%) among participants from Bulangshan. The respective prevalence rates in Pu'er were 66.9%, 56.5% and 9.2%. An infection with *Taenia *spp. was found in six participants from Bulangshan. Table 1 shows that soil-transmitted helminth infections were more prevalent among males than females and increased with age. Two-third of the boys (66.9%) had a hookworm infection, whereas half of the girls (51.2%) were infected with this parasite, accounting for a significant sex difference (*P *< 0.001). In Bulangshan, higher mean helminth egg counts were found than in Pu'er. With regard to infection intensity, as determined by the duplicate Kato-Katz thick smears and stratified according to WHO cut-offs [2], significant differences were found between the two schools. While in Pu'er only 2.1% of the students had a moderate/heavy infection intensity with *A. lumbricoides*, in Bulangshan, 36.4% were infected at this level of intensity (*P *< 0.001).

**Table 1:** Prevalences (pooled results from duplicate Kato-Katz and one FLOTAC test), and Kato-Katz-derived infection intensities of *A. lumbricoides*, hookworm and *T. trichiura *among 268 schoolchildren from two schools in Yunnan province, P.R. China, stratified by school, sex and age.

	Obs.	*A. lumbricoides*			Hookworm			*T. trichiura*		
				
		*N*	%	*P*-value	*N*	%	*P*-value	*N*	%	*P*-value
Total	268	125	46.6		160	59.7		134	50.0	
School										
Bulangshan	170	116	68.2	< 0.001	129	75.9	< 0.001	119	70.0	< 0.001
Pu'er	98	9	9.2		31	31.6		15	15.3	
Sex										
Female	123	49	39.8	0.040	63	51.2	0.009	52	42.3	0.020
Male	145	76	60.8		97	66.9		82	56.5	
Age (years)										
12-13	101	41	40.6	0.018	53	52.5	< 0.001	42	41.6	0.001
13-14	108	47	43.5		55	50.9		50	46.3	
> 14	59	37	62.7		52	88.1		42	71.2	

Infection intensity^a^										
Bulangshan										
Mean; 95% CI		6815	5069; 8563		600	367; 832		82	63; 120	
No		72	42.4		55	32.4		52	30.6	
Light		36	21.2		102	60.0		86	50.6	
Moderate		58	34.1		7	4.1		30	17.6	
Heavy		4	2.3		6	3.5		2	1.2	
Pu'er										
Mean; 95% CI		235	67; 537		175	74; 276		20	15; 33	
No		90	91.8		71	72.5		83	84.7	
Light		6	6.1		25	25.5		14.3	14	
Moderate		2	2.1		2	2.0		1	1.0	
Heavy		0	0		0	0		0	0	

All *P*-values calculated using χ^2 ^or Fisher's exact test as appropriate. ^a ^Stratification according to WHO [21].

Multiparasitism was pervasive in Bulangshan; almost 50% of the participants harboured three different helminth species concurrently (Table 2). In Pu'er, less than 10% of the participants were co-infected with two or more helminth species. Significant associations were observed between multiparasitism and both age (*P *< 0.001) and sex (*P *= 0.011), i.e. multiple species helminth infections were particularly prevalent among boys aged 14 years and above.

**Table 2:** Prevalence of multiple species helminth infections among 268 children from two schools in Yunnan province, P.R. China, stratified by school, sex and age.

		No infection		Single species infection		Dual species infection		Triple species infection	
					
	Obs.	*N*	%	*N*	%	*N*	%	*N*	%
All	268	68	25.4	60	22.4	59	22.0	81	30.2
School									
Bulangshan	170	15	8.8	24	14.1	51	30.0	80	47.1
Pu'er	98	53	54.1	36	33.7	8	8.2	1	1.0
Sex									
Female	123	40	32.5	28	22.8	29	23.6	26	21.1
Male	145	28	19.3	32	22.1	30	20.7	55	37.9
Age (years)									
12-13	101	34	33.7	26	25.7	13	12.9	28	27.7
13-14	108	31	28.7	26	24.1	25	23.1	26	24.1
> 14	59	3	5.1	8	13.5	21	35.6	27	45.8

Results obtained from duplicate Kato-Katz thick smears and a single FLOTAC test. All results were significant according to χ^2 ^or Fisher's test, as appropriate (*P *< 0.01).

### Association between QoL and helminth infections

The results obtained through the EQ-5D questionnaire are summarised in Table 3. Eight students were excluded from the analysis because they did not answer all questions. The seven (2.8%) participants who reported extreme problems in any of the EQ-5D dimensions were pooled with those reporting moderate problems.

**Table 3:** Number (%) of children reporting moderate or extreme problems on the EQ-5D questionnaire, stratified by helminth infection and school.

		Dimension											
		
		Mobility		Self-care		Usual activities		Pain or discomfort		Anxiety or depression		Any dimension	
							
Parasite	Obs.	*N *(%)	OR (95% CI)	*N *(%)	OR (95% CI)	*N *(%)	OR (95% CI)	*N *(%)	OR (95% CI)	*N *(%)	OR (95% CI)	*N *(%)	OR (95% CI)
*A. lumbricoides*													
Bulangshan													
Non-infected	48	11 (23)		3 (6)		20 (42)		39 (81)		29 (60)		41 (85)	
Infected	101	28 (28)	1.3 (0.6, 2.9)	10 (10)	1.6 (0.4, 6.3)	53 (52)	1.5 (0.8, 3.1)	69 (68)	0.5 (0.2, 1.1)	52 (51)	0.7 (0.3, 1.4)	90 (89)	1.4 (0.5, 3.9)
Pu'er													
Non-infected	89	3 (3)		3 (4)		36 (40)		53 (60)		59 (66)		74 (83)	
Infected	9	1 (11)	3.6 (0.3, 38.6)	0 (0)	n.a.	3 (33)	0.74 (0.2, 3.1)	7 (78)	2.4 (0.5, 12.1)	8 (89)	4.1 (0.5, 34.1)	9 (100)	n.a.
Hookworm													
Bulangshan													
Non-infected	36	10 (28)		3 (8)		20 (56)		29 (81)		20 (56)		32 (89)	
Infected	118	29 (26)	0.9 (0.4, 2.1)	10 (9)	1.0 (0.3, 4.1)	53 (47)	0.7 (0.3, 1.5)	79 (70)	0.6 (0.2, 1.4)	61 (54)	0.9 (0.4, 2.0)	99 (87)	0.9 (0.3, 2.9)
Pu'er													
Non-infected	67	3 (4)		2 (3)		20 (30)		37 (55)		43 (64)		55 (82)	
Infected	31	1 (3)	0.7 (0.1, 7.1)	1 (3)	1.1 (0.1, 12.4)	19 (61)	3.7 (1.5, 9.1)^a^	23 (74)	2.3 (0.9, 6.0)	24 (77)	1.9 (0.7, 5.1)	28 (90)	2.0 (0.5, 7.8)
*T. trichiura*													
Bulangshan													
Non-infected	46	10 (22)		4 (9)		24 (52)		35 (76)		31 (67)		41 (89)	
Infected	108	29 (26)	1.4 (0.6, 3.2)	9 (9)	1.0 (0.3, 3.4)	49 (47)	0.8 (0.4, 1.7)	73 (71)	0.8 (0.3, 1.7)	50 (49)	0.5 (0.2, 0.9)^a^	90 (87)	0.8 (0.3, 2.5)
Pu'er													
Non-infected	83	4 (5)		2 (2)		31 (37)		49 (59)		55 (66)		68 (82)	
Infected	15	0 (0)	n.a.	1 (7)	2.9 (0.2, 34.1)	8 (53)	1.9 (0.6, 5.8)	11 (73)	1.9 (0.6, 6.5)	12 (80)	2.0 (0.5, 7.8)	15 (100)	n.a

Odds ratios (OR) with 95% confidence intervals (CI) are presented. ^a ^Significant at *P *< 0.05 (χ^2 ^test), n.a., not applicable

Eighty-seven percent of the students reported to experience problems in at least one of the five dimension of the EQ-5D questionnaire. In less than two-thirds of all examined soil-transmitted helminth species combinations and EQ-5D dimension for both schools, students infected with one or several species of soil-transmitted helminths reported more problems than their non-infected peers. However, in the remaining one-third of the combinations, the opposite was observed, i.e. non-infected students reported more problems than those with a soil-transmitted helminth infection. Of note, the comparison between the two schools revealed important differences in some of the dimensions of the EQ-5D. 'Pain', for example, was reported by 68% of the *A. lumbricoides*-infected participants from Bulangshan, while 81% of the *A. lumbricoides*-negative students reported 'pain' (odds ratio (OR) = 0.5; 95% confidence interval (CI) = 0.2, 1.1). In contrast, in Pu'er, 78% of the participants with an *A. lumbricoides *infection reported 'pain', whereas the respective percentage among their non-infected counterparts was 60% (OR = 2.4; 95% CI = 0.5, 12.1). In Pu'er, 61% of the participants harbouring hookworms reported impairments in 'usual activities', whereas only 30% of the hookworm-free students reported impaired usual activity (OR = 3.7; 95% CI = 1.5, 9.1). The dimension 'mobility' was the only where a similar trend could be observed in both schools for children with an *A. lumbricoides *infection (Bulangshan: OR = 1.3; 95% CI = 0.6, 2.9; Pu'er: OR = 3.6; 95% CI = 0.3, 38.6). A counterintuitive trend in the same dimension was found for hookworm infection (Bulangshan: OR = 0.9; 95% CI = 0.4, 2.1; Pu'er: OR = 0.7; 95% CI = 0.1, 7.1).

Stratification by infection intensity and number of helminth species did not change the overall picture. Most problems were reported by students without any diagnosed helminth infection; the lowest number of self-reported impairment was often found among those with a single species helminth infection. With increasing infection intensity or number of helminth species, generally more impairments were reported. However, the differences were not statistically significant (data not shown). Participants from Pu'er had higher EQ-VAS scores than those from Bulangshan (Table 4). Only slight differences were observed between infected and non-infected students from either school. Participants from Bulangshan with moderate or heavy *A. lumbricoides *infection intensity had a borderline significant (*P *= 0.060) lower VAS score than non-infected participants.

**Table 4:** Mean EQ-VAS scores, including standard deviation (SD) and *A. lumbricoides*, hookworm and *T. trichiura *infection, together with the scores of the negative control groups among 252 children visiting two schools in Yunnan province, P.R. China.

Parameter	Obs.	Mean EQ-VAS score	SD	95% CI	Min. score	Max. score	***P*-value**^**a**^
*A. lumbricoides*							
Bulangshan							
Non-infected	49	66.4	19.8	60.7, 72.1	28	98	0.655
Infected	105	64.8	22.3	60.5, 69.2	0^b^	100	
Pu'er							
Non-infected	89	70.5	18.4	66.7, 74.4	18	100	0.777
Infected	9	68.3	19.0	53.7, 82.9	35	90	
Hookworm							
Bulangshan							
Non-infected	36	67.3	22.8	59.5, 75.0	0^b^	100	0.375
Infected	118	64.8	21.1	61.0, 68.6	25	100	
Pu'er							
Non-infected	67	70.5	18.5	66.0, 75.0	18	100	0.757
Infected	31	69.9	18.2	63.3, 76.6	39	100	
*T. trichiura*							
Bulangshan							
Non-infected	46	64.3	20.5	58.2, 70.3	27	94	0.787
Infected	108	65.8	22.0	61.6, 70.0	0^b^	100	
Pu'er							
Non-infected	83	69.7	18.9	65.6, 73.8	18	100	0.445
Infected	15	74.0	15.1	65.6, 82.3	50	100	

^a ^*P*-values according to the Kruskal-Wallis test with 1 d.f.

^b ^0 equals to death in EQ-VAS but some children presumably did not understand the question and answered 0.

CI, confidence interval; SD, standard deviation

The results of the SF-12 questionnaire are summarised in Table 5. Twelve participants were excluded from the analysis because they failed to answer all 12 questions. We could not find any differences between infected and non-infected participants in most dimensions, neither by comparing the mean health scores nor in the logistic regression model. According to the Kruskal-Wallis test, significant differences were found in 'role functioning' and 'PCS' among students from Pu'er, and 'mental health' among students from Bulangshan. In these domains, non-infected students had significantly lower mean health scores than their *A. lumbricoides-*infected ('role functioning' and 'PCS') or hookworm-infected ('mental health') counterparts. However, in the logistic regression, no differences (OR close to 1.0) were observed for these domains (data not shown).

**Table 5:** Mean health (standard deviation) score according to the SF-12 questionnaire among 238 children visiting two schools in Yunnan province, P.R. China, stratified by helminth infection (*A. lumbricoides*, hookworm, *T. trichiura*) and school.

Parameter	Obs.	Dimension									
		
		PF	RF	BP	GH	V	SF	RE	MH	PCS	MCS
*A. lumbricoides*											
Bulangshan											
Not infected	46	76 (24)	57 (41)	73 (20)	69 (27)	45 (27)	67 (27)	55 (42)	59 (18)	66 (26)	57 (25)
Infected	98	75 (24)	53 (40)	76 (16)	72 (26)	54 (28)	72 (23)	51 (42)	64 (19)	64 (25)	58 (25)
Pu'er											
Not infected	85	78 (23)	68 (40)^a^	69 (17)	72 (29)	62 (21)	82 (7)	33 (43)	68 (16)	56 (25)^a^	51 (24)
Infected	9	81 (24)	33 (43)	79 (16)	75 (23)	61 (22)	79 (18)	55 (43)	65 (18)	74 (26)	60 (26)
Hookworm											
Bulangshan											
Not infected	34	76 (24)	58 (40)	73 (20)	69 (26)	46 (27)	68 (26)	56 (41)	58 (18)^a^	67 (25)	57 (24)
Infected	110	74 (26)	47 (43)	76 (17)	74 (27)	54 (29)	69 (25)	46 (43)	69 (18)	61 (28)	57 (27)
Pu'er											
Not infected	64	73 (26)	63 (41)	75 (11)	73 (23)	56 (20)	77 (19)	50 (42)	62 (21)	68 (25)	56 (25)
Infected	30	84 (22)	65 (41)	79 (17)	75 (24)	64 (23)	80 (17)	55 (44)	67 (16)	74 (27)	61 (26)
*T. trichiura*											
Bulangshan											
Not infected	44	76 (24)	59 (40)	74 (21)	70 (27)	47 (28)	68 (27)	58 (41)	60 (19)	68 (25)	59 (25)
Infected	100	74 (25)	48 (42)	75 (14)	69 (27)	50 (26)	69 (23)	44 (42)	62 (19)	61 (27)	53 (24)
Pu'er											
Not infected	81	83 (21)	65 (43)	77 (16)	79 (20)	62 (21)	78 (15)	58 (49)	67 (15)	74 (30)	62 (26)
Infected	13	80 (24)	64 (41)	78 (16)	74 (24)	61 (23)	79 (18)	52 (43)	65 (19)	72 (26)	59 (26)

^a ^Significant at *P *< 0.05 (Kruskal-Wallis test with 1 d.f.)

PF, physical function; RF, role function; BP, bodily pain; GH, general health; V, vitality; SF, social functioning; RE, role emotional; MH, mental health; PCS, physical component summary; MCS, mental component summary

### Association between end-of-school-term marks and helminth infections

Table 6 summarises the associations between the end-of-school-term marks in Chinese, English, mathematics and sports and helminth infections. For each of the investigated soil-transmitted helminth infections, students without the respective infection had equal or slightly higher end-of-school-term marks when compared to their infected peers; differences were larger in Bulangshan than in Pu'er. Furthermore, a trend towards lower marks among those with heavier helminth infections was observed; mostly, however, without statistical significance. In mathematics, the participants from Bulangshan without a helminth infection had significantly higher marks (mean = 41 on a scale from 0 (lowest mark) to 100 (highest mark); standard deviation (SD) = 23) than participants with single (mean = 24, SD = 14), dual (mean = 21, SD = 13) or triple (mean = 24, SD = 17) helminth species infection, respectively (*P *= 0.007).

**Table 6:** Relationship between *A. lumbricoides*, hookworm and *T. trichiura *infection intensity, multiple helminth infection and school performance, including standard deviation (SD) measured by end-of-school-term marks (scale from 100 (best) to 0 (worst)) among 154 children from Bulangshan, Yunnan province, P.R. China.

Parameter	Obs.	End-of-school-term marks							
		
		Chinese		Mathematics		English		**Sports (n = 104)**^**c**^	
					
		Mean (SD)	***P*-value**^**a**^	Mean (SD)	*P*-value	Mean (SD)	*P*-value	Mean (SD)	*P*-value
*A. lumbricoides*									
No^b^	66	46 (19)	0.107	26 (17)	0.342	42 (18)	0.600	78 (12) (n = 47)	0.130
Light	33	38 (19)		21 (15)		38 (23)		75 (12) (n = 20)	
Moderate	51	42 (19)		25 (18)		44 (19)		73 (14) (n = 34)	
Heavy	4	32 (13)		20 (13)		37 (23)		55 (23) (n = 3)	
Hookworm									
No	49	46 (20)	0.110	29 (18)	0.090	42 (21)	0.586	75 (11) (n = 34)	0.321
Light	95	42 (19)		23 (16)		42 (18)		75 (15) (n = 63)	
Moderate	5	44 (15)		20 (9)		31 (22)		81 (9) (n = 4)	
Heavy	5	25 (7)		15 (8)		48 (28)		65 (5) (n = 3)	
*T. trichiura*									
No	47	47 (20)	0.360	29 (19)	0.126	42 (15)	0.326	77 (15) (n = 34)	0.438
Light	78	41 (18)		23 (15)		39 (21)		75 (13) (n = 48)	
Moderate	27	40 (17)		21 (13)		44 (22)		74 (12) (n = 21)	
Heavy	2	48 (53)		39 (47)		60 (0)		90 (n.a.) (n = 1)	
Multiple helminth infection									
No	13	54 (21)	0.089	41 (23)	0.007	45 (19)	0.697	80 (11) (n = 10)	0.773
Single	22	47 (18)		24 (14)		41 (17)		76 (12) (n = 17)	
Dual	46	43 (20)		21 (13)		39 (17)		75 (15) (n = 31)	
Triple	73	40 (18)		24 (17)		43 (22)		74 (13) (n = 46)	

^a ^*P*-values according to the Kruskal-Wallis test with 3 d.f. ^b ^Stratification according to WHO [21]. ^c ^One class had no sports education

## Discussion

Prior to our study, the two standardised questionnaires EQ-5D and SF-12 had been employed in P.R. China to estimate disability weights for chronic schistosomiasis japonica [15] and echinococcosis [16]. We have now made an attempt of using EQ-5D and SF-12 for the appraisal of self-rated QoL in relation to soil-transmitted helminth infections. The results obtained from the two QoL questionnaires and their associations with students' parasitological findings are not easy to interpret and setting-specific idiosyncrasies were observed. Indeed, in only two out of 15 examined associations (five dimensions of EQ-5D each for *A. lumbricoides*, hookworm and *T. trichiura*) we found a trend into the same direction for both schools, with more reported problems among helminth-infected compared to non-infected students ('mobility' in relation to *A. lumbricoides*) or more reported problems among the non-infected ('mobility' in relation to hookworm), respectively. For the remaining 13 associations, no clear trends were observed, i.e. if in one dimension helminth-infected students from Pu'er reported more impairments than their non-infected peers, then in Bulangshan more impairments were reported among the non-infected students. There was a tendency of non-infected individuals from Bulangshan reporting more problems than their helminth-infected counterparts. In contrast to previous studies, there was no difference in the sensitivity of SF-12 and EQ-5D [23]. Finally, logistic regression modelling suggested that there were no differences between the self-rated QoL among helminth-infected and non-infected participants.

Interestingly, clearer trends were observed between the end-of-school-term marks and helminth infections. Although most of the results were not significant, we could document a trend towards lower marks in Chinese, English and mathematics as the infection intensity or the number of helminth species increased. These results are in agreement with previous studies [24-26] and confirm the notion that helminth infections may negatively impact on students' cognitive skills.

Considering the parasitological results, previous studies in Yunnan province had revealed that soil-transmitted helminth infections and multiparasitism were common among ethnic minorities, including the Bulang [17,27,28]. The significantly lower helminth prevalences found in peri-urban Pu'er can be explained by the yearly school-based deworming campaigns implemented over the past years as well as the generally better socio-economic conditions in this city compared to Bulangshan.

Our study has several drawbacks, which are offered for discussion. Firstly, the overall sample size (final cohort: 252 individuals) was small. Secondly, only a single stool sample was subjected to duplicate Kato-Katz thick smears and a single FLOTAC. Were multiple stool samples examined with additional diagnostic approaches, it is conceivable that more infections would have been detected, most notably of light intensity [17,29,30]. Thirdly, a one-to-one administration of the questionnaire was not feasible, yet efforts were made to ascertain quality data. One staff member read the questions aloud in front of 20-30 students and answered upcoming questions. After the questionnaire survey, the filled-in forms were carefully reviewed for missing or implausible answers and, when need be, the questionnaire was completed together with the child. It should be noted that one-to-one administration also holds risks, namely of suggesting seemingly "right" answers. Fourthly, we had the impression that the two questionnaires administered concurrently were too long for the students and that attention was diverted over the course of filling in the questionnaire. During the pre-test of the questionnaire in a nearby village, no similar problems were observed.

It is also important to note that the two selected schools were indeed quite different. While the school in Bulangshan is a rural boarding school in a very poor and remote district, the second school is situated on the outskirts of Pu'er, a prefecture-level city with more than 100,000 inhabitants. It is conceivable that these contextual determinants govern the students' perception of health and might go a long way to explain the observed differences in self-rated QoL between the two schools. In a study carried out in Côte d'Ivoire, Raso and colleagues noted differences in perceived ill-health among the poorest and less poor schoolchildren [31]. The authors speculated that students from households with a higher socio-economic status might have higher expectations for their health, and hence are more sensitive about distress. Children who have a history of illness experience might have lower expectations and ignore symptoms. However, in a comparison of the main sources of family income, no correlation with self-rated QoL was found in the present study. This would be in agreement with an investigation from Sweden which analysed whether inequality in self-reported health predict inequality in socio-economic status and came to the conclusion that sex and age, but not income or educational attainment, have an effect on self-rated QoL [32]. More research is therefore needed to investigate whether or not socio-economic status impacts on QoL.

Our results also raise questions whether the chosen questionnaires are appropriate for this specific socio-cultural setting and the age group interviewed here. We gained the impression that some of the students had difficulties to fully apprehend some of our questions, most notably the EQ-VAS tool, and some general questions proved difficult for the participants to readily answer. In some instances, participants failed to understand questions even after face-to-face explanation. This might explain how a score of '0' in EQ-VAS, which equals to 'worst imaginable health state', or death, and was indicated as such on the form, could be reported.

It is important to note that students participating in the current study are likely to suffer from health issues other than helminth infections. The effects of co-infections and co-morbidities may predominate over the mainly subtle effects of soil-transmitted helminth infections. Both EQ-5D and SF-12 only measure the overall QoL, and attribution of results to specific diseases is impossible. Furthermore helminth infections are of chronic nature [33] and nearly ubiquitous, particularly in Bulangshan, and hence might force children to adapt and perceive their effects as normal. End-of-school-term marks, on the other hand, more readily capture the longitudinal effects of soil-transmitted helminth infections and other chronic conditions, as shown here. Furthermore, it has been reported that QoL questionnaires can show ceiling effects, i.e. patients with the best score in self-rated QoL may have substantial impairments as measured by more objective tests. On the other hand, the tools used here may also have floor effects, i.e. patients with the worst score in self-rated QoL may deteriorate even further [34].

## Conclusions

Unresolved issues and challenges have been identified in the present study when attempting to relate helminth infections with self-rated QoL. Seemingly more meaningful results pertaining to the effects of soil-transmitted helminth infections on children were obtained when using simple end-of-school-term marks rather than sophisticated tools such as EQ-5D and SF-12 questionnaires. However, since school marks are not readily comparable across schools, we conclude that further development of QoL instruments is warranted with a view towards further adopting them to target populations. To do so, it is necessary to better understand local concepts regarding health and perceived morbidity.

## Competing interests

The authors declare that they have no financial, professional or personal competing interests related to this article. The funding agencies played no role in the design or implementation of the study, analysis or interpretation of the data, or the preparation and submission of the manuscript.

## Authors' contributions

KZ designed and implemented the study, managed, analysed and interpreted the data and prepared the manuscript; PS designed the study, facilitated and assisted the study implementation, interpreted the data and revised the manuscript; HZ, ZWD, JYJ and TWJ assisted in the design and study implementation and revised the manuscript; TF assisted in the design of the study, data analysis and interpretation and revision of the manuscript; XNZ designed the study, supervised the study implementation and revised the manuscript; JU designed the study, supervised KZ, interpreted the data and revised the manuscript. All authors read and approved the final manuscript.

## References

[B1] Murray CJL Lopez AD Global burden of disease. Comprehensive assessment of mortality and disability from diseases, injuries, and risk factors in 1990 and projected to 2020 1996 Cambridge, MA: Harvard School of Public Health

[B2] WHO Prevention and control of schistosomiasis and soil-transmitted helminthiasis: report of a WHO expert committee WHO Tech Rep Ser 2002 912 1 57 12592987

[B3] Chan MS The global burden of intestinal nematode infections - fifty years on Parasitol Today 1997 13 438 443 10.1016/S0169-4758(97)01144-7 15275146

[B4] Anand S Hanson K Disability-adjusted life years: a critical review J Health Econ 1997 16 685 702 10.1016/S0167-6296(97)00005-2 10176779

[B5] Mathers CD Ezzati M Lopez AD Measuring the burden of neglected tropical diseases: the global burden of disease framework PLoS Negl Trop Dis 2007 1 e114 10.1371/journal.pntd.0000114 18060077 PMC2100367

[B6] Bethony J Brooker S Albonico M Geiger SM Loukas A Diemert D Hotez PJ Soil-transmitted helminth infections: ascariasis, trichuriasis, and hookworm Lancet 2006 367 1521 1532 10.1016/S0140-6736(06)68653-4 16679166

[B7] Dickson R Awasthi S Demellweek C Williamson P Anthelmintic drugs for treating worms in children: effects on growth and cognitive performance Cochrane Database Syst Rev 2000 CD000371 10796537 10.1002/14651858.CD000371

[B8] King CH Bertino AM Asymmetries of poverty: why global burden of disease valuations underestimate the burden of neglected tropical diseases PLoS Negl Trop Dis 2008 2 e209 10.1371/journal.pntd.0000209 18365036 PMC2267491

[B9] Guyatt GH Feeny DH Patrick DL Measuring health-related quality of life Ann Intern Med 1993 118 622 629 8452328 10.7326/0003-4819-118-8-199304150-00009

[B10] Guyatt GH Veldhuyzen van Zanten SJ Feeny DH Patrick DL Measuring quality of life in clinical trials: a taxonomy and review CMAJ 1989 140 1441 1448 2655856 PMC1269981

[B11] EuroQoL EuroQol - a new facility for the measurement of health-related quality of life Health Policy 1990 16 The EuroQol Group 199 208 10.1016/0168-8510(90)90421-9 10109801

[B12] Stewart AL Ware JE Measuring functions and well-being: the medical outcomes study approach 1992 Durham, NC: Duke University Press

[B13] Wang H Kindig DA Mullahy J Variation in Chinese population health related quality of life: results from a EuroQol study in Beijing, China Qual Life Res 2005 14 119 132 10.1007/s11136-004-0612-6 15789946

[B14] Lam CL Tse EY Gandek B Is the standard SF-12 health survey valid and equivalent for a Chinese population? Qual Life Res 2005 14 539 547 10.1007/s11136-004-0704-3 15892443

[B15] Jia TW Zhou XN Wang XH Utzinger J Steinmann P Wu XH Assessment of the age-specific disability weight of chronic schistosomiasis japonica Bull World Health Organ 2007 85 458 465 10.2471/BLT.06.033035 17639243 PMC2636356

[B16] Budke CM Jiamin Q Zinsstag J Qian W Torgerson PR Use of disability adjusted life years in the estimation of the disease burden of echinococcosis for a high endemic region of the Tibetan plateau Am J Trop Med Hyg 2004 71 56 64 15238690

[B17] Steinmann P Du ZW Wang LB Wang XZ Jiang JY Li LH Marti H Zhou XN Utzinger J Extensive multiparasitism in a village of Yunnan province, People's Republic of China, revealed by a suite of diagnostic methods Am J Trop Med Hyg 2008 78 760 769 18458311

[B18] Steinmann P Zhou XN Du ZW Jiang JY Wang LB Wang XZ Li LH Marti H Utzinger J Occurrence of *Strongyloides stercoralis *in Yunnan province, China, and comparison of diagnostic methods PLoS Negl Trop Dis 2007 1 e75 10.1371/journal.pntd.0000075 17989788 PMC2041812

[B19] Katz N Chaves A Pellegrino J A simple device for quantitative stool thick-smear technique in schistosomiasis mansoni Rev Inst Med Trop São Paulo 1972 14 397 400 4675644

[B20] Cringoli G Rinaldi L Maurelli MP Utzinger J FLOTAC: new multivalent techniques for qualitative and quantitative copromicroscopic diagnosis of parasites in animals and humans Nat Protoc 2010 5 503 515 10.1038/nprot.2009.235 20203667

[B21] Montresor A Crompton DWT Hall A Bundy DAP Savioli L Guidelines for the evaluation of soil-transmitted helminthiasis and schistosomiasis at community level 1998 Geneva: World Health Organization

[B22] Ware JE Kosinski M Turner-Bowker DM Gandek B How to score version 2 of the SF-12 health survey (with a supplement documenting version 1) 2002 Lincoln, RI: QualityMetric Inc

[B23] Johnson JA Pickard AS Comparison of the EQ-5D and SF-12 health surveys in a general population survey in Alberta, Canada Med Care 2000 38 115 121 10.1097/00005650-200001000-00013 10630726

[B24] Dickson R Awasthi S Williamson P Demellweek C Garner P Effects of treatment for intestinal helminth infection on growth and cognitive performance in children: systematic review of randomised trials BMJ 2000 320 1697 1701 10.1136/bmj.320.7251.1697 10864543 PMC27412

[B25] Grigorenko EL Sternberg RJ Jukes M Alcock K Lambo J Ngorosho D Nokes C Bundy DAP Effects of antiparasitic treatment on dynamically and statically tested cognitive skills over time J Appl Dev Psycho 2006 27 499 526 10.1016/j.appdev.2006.08.005

[B26] Ezeamama AE Friedman JF Acosta LP Bellinger DC Langdon GC Manalo DL Olveda RM Kurtis JD McGarvey ST Helminth infection and cognitive impairment among Filipino children Am J Trop Med Hyg 2005 72 540 548 15891127 PMC1382476

[B27] Steinmann P Zhou XN Du ZW Jiang JY Xiao SH Wu ZX Zhou H Utzinger J Tribendimidine and albendazole for treating soil-transmitted helminths, *Strongyloides stercoralis *and *Taenia *spp.: open-label randomized trial PLoS Negl Trop Dis 2008 2 e322 10.1371/journal.pntd.0000322 18923706 PMC2561005

[B28] Steinmann P Utzinger J Du ZW Zhou XN Multiparasitism: a neglected reality on global, regional and local scale Adv Parasitol 2010 73 21 50 full_text 20627138 10.1016/S0065-308X(10)73002-5

[B29] Booth M Vounatsou P N'Goran EK Tanner M Utzinger J The influence of sampling effort and the performance of the Kato-Katz technique in diagnosing *Schistosoma mansoni *and hookworm co-infections in rural Côte d'Ivoire Parasitology 2003 127 525 531 10.1017/S0031182003004128 14700188

[B30] Knopp S Mgeni AF Khamis IS Steinmann P Stothard JR Rollinson D Marti H Utzinger J Diagnosis of soil-transmitted helminths in the era of preventive chemotherapy: effect of multiple stool sampling and use of different diagnostic techniques PLoS Negl Trop Dis 2008 2 e331 10.1371/journal.pntd.0000331 18982057 PMC2570799

[B31] Raso G Utzinger J Silué KD Ouattara M Yapi A Toty A Matthys B Vounatsou P Tanner M N'Goran EK Disparities in parasitic infections, perceived ill health and access to health care among poorer and less poor schoolchildren of rural Côte d'Ivoire Trop Med Int Health 2005 10 42 57 10.1111/j.1365-3156.2004.01352.x 15655013

[B32] van Doorslaer E Gerdtham UG Does inequality in self-assessed health predict inequality in survival by income? Evidence from Swedish data Soc Sci Med 2003 57 1621 1629 10.1016/S0277-9536(02)00559-2 12948571

[B33] King CH Dangerfield-Cha M The unacknowledged impact of chronic schistosomiasis Chronic Illn 2008 4 65 79 10.1177/1742395307084407 18322031

[B34] Eiser C Morse R Quality-of-life measures in chronic diseases of childhood Health Technol Assess 2001 5 1 157 11262421 10.3310/hta5040

